# Recent Advances in Neural Recording Microsystems

**DOI:** 10.3390/s110504572

**Published:** 2011-04-27

**Authors:** Benoit Gosselin

**Affiliations:** Electrical and Computer Engineering Department, Université Laval, 1065 avenue de la Médecine, Québec (Québec) G1V 0A6, Canada; E-Mail: benoit.gosselin@gel.ulaval.ca; Tel.: +1-418-656-2131; Fax: +1-418-656-3159

**Keywords:** implantable microsystems, neural recording, brain-computer interfaces, sensory circuits, data management, power scheduling, low-power biotelemetry, multi-channel, inductive link, ultrawide-band

## Abstract

The accelerating pace of research in neuroscience has created a considerable demand for neural interfacing microsystems capable of monitoring the activity of large groups of neurons. These emerging tools have revealed a tremendous potential for the advancement of knowledge in brain research and for the development of useful clinical applications. They can extract the relevant control signals directly from the brain enabling individuals with severe disabilities to communicate their intentions to other devices, like computers or various prostheses. Such microsystems are self-contained devices composed of a neural probe attached with an integrated circuit for extracting neural signals from multiple channels, and transferring the data outside the body. The greatest challenge facing development of such emerging devices into viable clinical systems involves addressing their small form factor and low-power consumption constraints, while providing superior resolution. In this paper, we survey the recent progress in the design and the implementation of multi-channel neural recording Microsystems, with particular emphasis on the design of recording and telemetry electronics. An overview of the numerous neural signal modalities is given and the existing microsystem topologies are covered. We present energy-efficient sensory circuits to retrieve weak signals from neural probes and we compare them. We cover data management and smart power scheduling approaches, and we review advances in low-power telemetry. Finally, we conclude by summarizing the remaining challenges and by highlighting the emerging trends in the field.

## 1. Introduction

For several decades, researchers have elaborated various types of experimental setups for studying the central nervous system (CNS) at the cellular level. Sixty years ago, Hodgkin and Huxley [1] performed intracellular recordings using glass capillary electrodes filled with seawater to characterize the squid giant axon. Such experiments allowed the understanding and the precise modeling of the generation of action potentials by neurons. During the 50s and the 60s, single cell (single unit) recordings performed with extracellular metal microelectrodes by Mountcastle [2] and Hubel [3] led to fundamental discoveries about the structure and the organization of the cerebral cortex. Such recordings helped in establishing the columnar organization of the visual cortex and allowed the understanding of the importance of biopotentials in the transmission of information in the CNS. The research carried out by several neuroscientists in the 80s revealed the correlation between limb movement and neural activity in many motor areas of the cortex. Georgopoulos demonstrated that while single unit recordings correlate poorly with hand motion, the combined activity of several neurons conveys the precise direction of movement [4]. Such results have generated a great interest in the simultaneous recording of the activity of several individual neurons in the cortex, *i.e*., multi-unit recordings. Since then, the multi-unit approach has been extensively used in various neuroscience research applications [5]. The 90s have seen a sustained research effort directed towards extracting neural signals and using them to control various prosthetics devices. Experimentation with non-human primates allowed characterization of the simultaneous firing patterns of tens of cells associated with limb movements in a three dimensional space [6], and the subsequent use of this neural activity to move a cursor on a computer screen or control a robot arm [7]. The characterization of hand trajectory has also been investigated [8], and the potential of neural prosthetic devices for helping paralyzed humans has also been clearly demonstrated [9].

Nowadays, neural interfacing microsystems capable of continuously monitoring large groups of neurons are being actively researched by leveraging the recent advancements in neurosciences, microelectronics, communications and microfabrication. Such microsystems are intended to directly tap into the source of voluntary control, the CNS, to feed prosthetic devices restoring or replacing sensory, motor, or cognitive functions. Cochlear implants and retinal stimulators [10] are excellent examples of successful neural interfacing technologies that have reached widespread clinical applications. Implantable neural microdevices are pursuing two critical objectives: (1) replacing hardwired connections with a wireless link to eliminate cable tethering and risks of infection, (2) enabling the local processing of neural signals on a chronic basis to avoid noise sources and improve signal integrity. Indeed, neural interfacing technologies that are specifically addressing research purposes do not necessarily need to be fully implantable, but they should provide high quality of data while eliminating hardwires to enable experiments with animal models that are free from any constraint of movement.

A suitable interface to the cortex must enable chronic utilization and high-resolution by the simultaneous sampling of the activity of hundreds of neurons. Neural microsystems must feature several data acquisition channels, each featuring low-noise amplifiers, filters, data converters, biopotential detectors, wireless transmitters and receivers, and power management blocks. However, the requirement of eventual implantability places severe limitations on size, weight, power consumption and bandwidth. Embedded circuits must dissipate very low power in order to limit heat and temperature rises in the surrounding tissues, but since low-power telemetry usually comes with narrower bandwidths, achieving intensive throughputs is difficult. Indeed, designing a telemetry unit for handling hundreds of independent channels, each generating around 0.3 Mbit/s (considering 10 bit per sample and a typical sampling frequency of 30 kHz per channel) while addressing small size, low-power and electromagnetic compatibility is very challenging. A significant research effort is presently devoted to increasing the power efficiency in neural microsystems through the development of low-power circuit techniques, efficient data management, smart power scheduling and the design of novel low-power telemetry modules. Indeed, data management techniques are researched for efficiently selecting only the relevant portions in the measured input signal and achieving manageable data rates. The design of energy-efficient circuits, smart on-chip powers saving approaches and low-power transmitters is fundamental in order to meet the requirements while providing high-density in this application.

The implementation of neural recording microsystems is multi-disciplinary since it involves several aspects such as electrodes, materials, interconnects, electronics and system integration. The growing impact and the high significance of neural interfacing technology in multiple areas of science are demonstrated by the sustained number of works that are presented at international events and in dedicated publications [11,12]. Moreover, several exhaustive reviews covering this discipline have been published [13–16]. This paper presents the latest advances in neural recording technology with a focus on the physiological requirements, and the design of the recording and telemetry electronics. The paper is organized as follows: Section 2 reviews the different types of neural signal modalities and Section 3 presents the main microsystem interfacing approaches. Section 4 discusses several sensory circuit topologies for interfacing with various types of neural probes. Sections 5 and 6 survey on-chip data management strategies and smart power scheduling approaches. Section 7 covers the latest advances in low-power biotelemetry. Finally, conclusions are drawn and some future directions are suggested in the last section.

## 2. Neural Probes and Signals Modalities

There is a manifold of neural signal modalities that convey useful information in the CNS, both in the electrical and in the chemical realm. The electroencephalogram (EEG) consists of biopotentials that can be measured un-invasively on the scalp using surface electrodes. However, most effective approaches for the direct control of prosthetic devices consist of capturing the signal inside the body using implantable electrodes [14]. Invasive bioelectrical and neurochemical recordings are performed inside the body with sharp biocompatible microelectrodes.

### 2.1. Bioelectrical Recording

The bioelectrical activity that is transmitted along the axon of a neuron can either be measured intracellularly or extracellularly using a microelectrode. The depolarisations of the membrane of a neuron generate extracellular action potentials (AP), which are waveforms in the range of 100 Hz to 10 kHz, whose duration is of a few milliseconds. The rate of occurrence of APs varies from 10 to 120 per second. Intracellular APs have amplitudes in the range of 70 mVpp [17], whereas extracellular APs have weak amplitudes ranging from 50 μVpp to 500 μVpp, depending on the distance between the active neuron and the recording electrode [18]. Intracellular APs can be measured by penetrating the membrane of a neuron with a sharp glass micropipette filed with saline water. A metal electrode in contact with the electrolyte connects the pipette to a circuit for amplification and interfacing with computers. Such an approach allows establishing a very good electrical connection by measuring strong voltage signals. However, it causes the death of the cell within a few hours after the recordings. Another type of biopotentials known as local field potential (LFP) consists of lower-frequency neural waveforms (mHz to 200 Hz) having amplitudes ranging from 500 μVpp to 5 mVpp. LFPs are also recorded using extracellular microelectrodes. They likely contain important complementary information on neural network dynamics and are useful in brain interfacing applications [19,20].

Patch clamp recording allows measuring current flowing from a single or a few ion channels by creating a sealed area on the surface of a cell membrane and the tip of a glass micropipette. This technique is less invasive than intracellular recording, but has also a negative impact on the cell since a part of the cellular membrane is destroyed after removing the seal. Integrated patch clamp amplifiers have been demonstrated [21]. However, both intracellular microelectrodes and patch clamping have limited potential for chronic and implantable technologies. Indeed, they do not allow simultaneous recordings from several individual cells, and they are not suitable for long-term studies or chronic settings, but they are useful for *in-vitro* measurements involving cells grown in dishes. In contrast, the monitoring of the activity of large groups of neurons for long periods of time is reported by several researchers [22], which demonstrate the high potential of such an approach for chronic applications. The most widespread and simpler implantable electrode technologies are metal wire bundles and microfabricated electrode arrays. The design and the fabrication of such probes are covered in [23].

### 2.2. Neurochemical Recording

On the other hand, measuring the neurochemical activity is an important milestone because the action of neurotransmitters is complementary to electrophysiology. The release or uptake of neurotransmitters establishes a conduction path between synapses, and determines the probability of whether a new action potential will be generated. Moreover, the measurement of neurotransmitters, such as dopamine and nitric oxide, can provide invaluable knowledge for the understanding of neurological disorders, like epilepsy and Parkinson’s disease. Neurotransmitters can be measured employing carbon fiber [24,25] or iridium oxide microelectrodes [26] by using amperometry or voltammetry. Indeed, fast-scan cyclic voltammetry is often preferred due to its shorter time scale allowing the monitoring of electro-chemical species in real time. Electrochemical analysis is based on the fact that certain species can undergo a chemical reaction on the surface of an electrode held at a characteristic potential, the redox potential. An electron-transfer reaction taking place between the species and the electrode generates a redox current that can be measured with three electrodes and a sensitive circuit. The redox potential is applied between a working electrode and a reference electrode, while the redox current flows from a counter electrode towards the working electrode. Knowing the preferred redox potential and redox current associated with a species enables to identify the neurochemical molecule and its concentration.

### 2.3. Summary of Recording Parameters

Table 1 summarizes the neural recording parameters for the different signal modalities. It is shown that intracellular AP measurements deal with strong signals, but have very low potential for chronic utilization, whereas extracellular AP and LFP measurements deal with weak signals, but have high potential for chronic utilization. The measurement of EEG has similar parameters then for LFPs, however measuring the latter is highly invasive compared to the former. Besides, chemical measurements involve very small redox currents, but offer high potential for chronic utilization. In contrast, patch-clamping measurements deal with larger currents but have rather low potential for chronic utilization.

## 3. Multi-Channel Recording System Architectures

Different types of neural microsystem architectures have been demonstrated for interfacing with multi-channel neural probes. Devices featuring hundreds of recording channels have been demonstrated [27–29]. Application specific integrated circuits (ASIC) can be interconnected with neural probes in different ways to form self-contained devices. Requirements on form factors imply that the IC must ideally accommodate a silicon area as low as 0.160 mm^2^ per channel because a 400-μm inter-electrode spacing is commonly used for good spatial resolution in the array. Devices integrating processing electronics on the same substrate as a silicon microelectrode array are made using IC fabrication technology [16]. Such processes allow construction of three-dimensional arrangements by assembling several planar parts using sophisticated techniques. Hybrid devices have been built using flip-chip approaches where one IC is connected to a silicon microelectrodes array [28,29]. This strategy offers high flexibility because the array and the mounted components (IC and passive parts) can be fabricated separately. This monolithic configuration is intended to be placed under the skull, in direct contact with the cerebral cortex, like a single intra-cortical button. Techniques using polymer films are used for the assembly of a probe with ASICs and with other mounted components on the same substrate [30]. Although such an approach enables achieving more complexity, it can result in bulky devices that may not be suitable for full implantability. Moreover, the horizontal assembly of several components can prohibit integration of a system within a prescribed area. Indeed, large surfaces that can be in direct contact with cortical tissues for lengthy periods can induce harmful effects such as inflammations and cortical œdema [31]. The approaches proposed in [32,33] circumvent such drawbacks by means of distributed configurations consisting of a front-end intra-cortical device that is interfaced with a cortically inserted probe, which is hardwired to a back-end cranial module atop of the skull. While such an approach enables relaxing constraints on the back-end module, any tethering in the interconnections could be harmful, and needs therefore to be addressed. The vertical integration of a multi-chip microsystem for increasing the complexity of an intra-cortical button-type neural microsystem along the z-axis is proposed in [31]. The interaction with the aforementioned tissue layers are therefore minimized in this configuration, given that the height of the stacked structure is kept within the intracranial space (2–3 mm) to limit any rise of the implant above the cortex and provide clearance.

**Figure 1.**
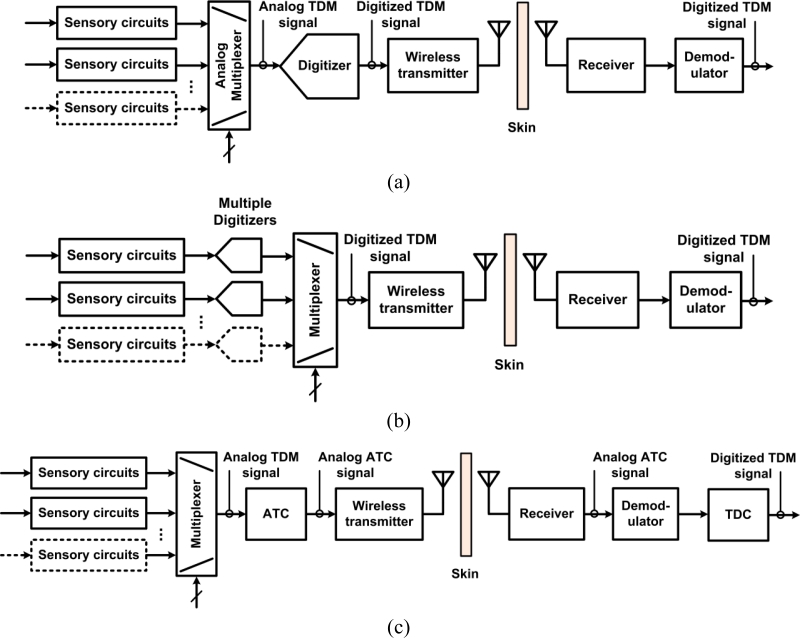
Block diagrams showing different neural recording microsystem architectures. **(a)** This approach is employing analog TDM and is sharing a fast ADC between channels. **(b)** This approach is employing digital TDM and is using one ADC per channel. **(c)** This approach is employing analog TDM and is transferring digitization on the remote station side by using ATC in the implanted part, followed by TDC in the external part.

The recording electronics in the ASICs are based on different system-level approaches. Figure 1 presents three different neural microsystem system-level architectures. A widespread approach consists in sharing a fast digitizer between the multiple sensory channels. The sensory channels are directed toward the analog-to-digital-converter (ADC) employing time-division multiplexing (TDM) in the analog domain. The challenge with such an approach is how to minimize power consumption from the ADC and from the several unity-gain buffers needed to drive the ADC at high rate and isolate channels from each other. Moreover, the analog multiplexer must be designed carefully in order to avoid excessive crosstalk. An example of such a type of implementation is presented in [32]. Another approach consists in providing one low-power low-rate ADC for each sensory channel and performing TDM in the digital domain instead. The main benefits of such an approach are that it avoids the need for several power-consuming unity gain buffers and it eliminates inter-channel crosstalk. However, great care must be taken in the design of the ADC in order to minimize the chip area. Such a type of implementation is presented in [31]. A third approach consists in performing digitization off the chip to save power and chip area. Digitization is performed in two phases. A first phase consists of converting the multiplexed analog sensory output to time duration, known as analog-to-time conversion (ATC). Then, such an ATC signal is transmitted outside the body, to a remote base station where power and size are not so highly constrained for performing time-to-digital conversion (TDC). This approach requires performing TDM in the analog domain first, which promotes crosstalk, but it has the advantage of not requiring any clock signals. Such an approach is presented in [34].

Besides, implantable microsystems dedicated to the measurement and the detection of neurotransmitters from single or multiple electrodes have been demonstrated [25,35,36]. Electrochemical measurements from multiple electrodes are very attractive because they offer a distributed view of the release of several types of highly diffusive neurotransmitters. Moreover, a two-electrode electro-analysis approach is normally employed in a neurochemical recording microsystem format since the very small redox current involves negligible ohmic drop in the reference electrode. Thus, the reference electrode is commonly being short-circuited to the counter electrode [24,25].

## 4. Neural Sensory Circuits

Extracellular electrodes generate weak analog signals that need to be amplified, filtered and digitized to be transferrable to the world of computing for further processing and storage. Such functions are conducted in dedicated circuits often referred to as the analog front-end. The low-noise amplifier (LNA) is the main building block of such front-ends. Also referred to as a neural amplifier, the LNA must amplify and filter the neural waveforms in order to remove any DC offset seen across a pair of differential electrodes. Indeed, it must provide sufficient gain, appropriate bandwidth, high signal-to-noise ratio (SNR), excellent linearity, high common mode and power supply rejection ratios (CMRR and PSRR), in order to provide the required signal quality. In the case of a multi-channel interface, one such sensory circuit is needed for each electrode. Therefore, a neural amplifier must present low-power and small size, and be scalable to multiple parallel channels. Furthermore, it is essential to optimize the design for very low-power operation through a dedicated circuit design methodology, like in [37]. The noise efficiency factor (NEF) has been widely adopted as a main figure of merit to assess the performance of neural amplifiers and compare the several existing topologies together. Most effective neural amplifier designs achieving NEF values between 2 and 6.

**Figure 2.**
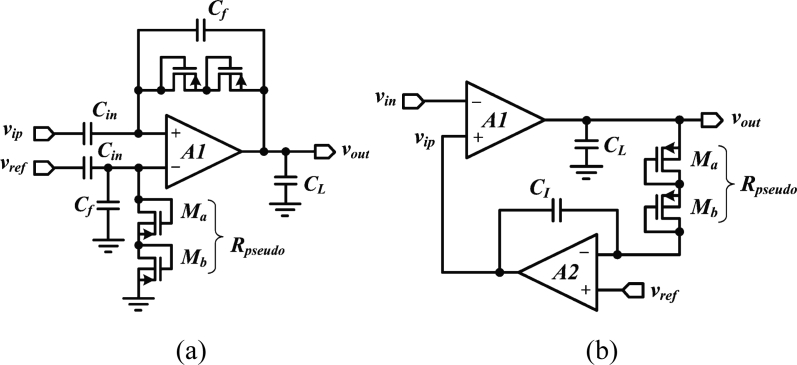
**(a)** Schematic of a capacitive-feedback neural amplifier. **(b)** Schematic of an active-feedback neural amplifier.

In addition to low-noise and low-power optimization, suppressing the DC input voltage while leaving the broadband neural signal (from 1 mHz to 10 kHz) untouched represents a considerable circuit design challenge in an integrated device, because it requires the implementation of a very low-cutoff frequency. Except for specific designs that address extremely low-power [38] or very-low-noise requirements [39], the design of reliable integrated neural amplifiers has progressed from opened-loop to closed-loop topologies. Early approaches consist in loading the recording site with a high-value resistor, implemented either with a reverse-biased diode [13], or with subthreshold transistors forming a highpass filter with the electrode capacitance [40]. In present devices, closed-loop DC suppression schemes have replaced opened-loop approaches. A popular approach, depicted in Figure 2(a), consists of forming a closed-loop gain along with a low-frequency pole using a capacitive feedback networks and a highly resistive MOS-bipolar element [37]. Since the equivalent resistor of a MOS-bipolar element can reach several thousands of giga-Ohms for small voltages, it is sufficient to use a small integrated capacitors of a few tens of pico-Farad in the feedback network for implementing a low-frequency cutoff that is in the mHz range. The feedback loop is normally built around a low-noise operational transconductor amplifier (OTA) which implementation varies.

**Figure 3.**
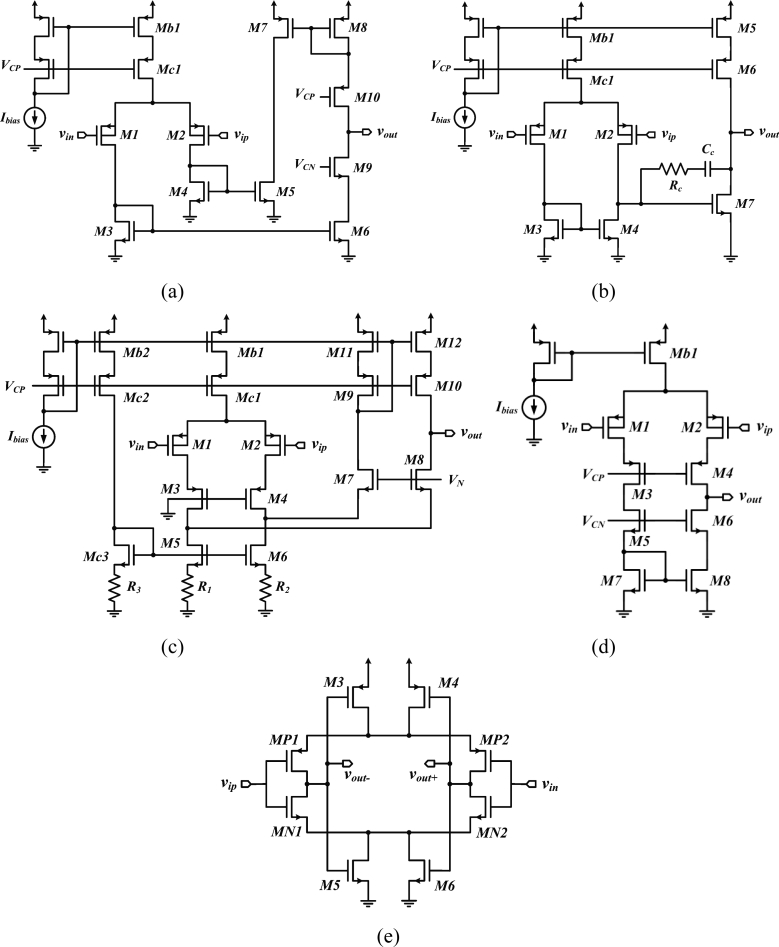
**(a)** Current-mirror OTA. **(b)** Miller OTA. **(c)** Folded OTA with source degeneration. **(d)** Telescopic OTA. **(e)** Self-biased OTA.

The current mirror OTA [34,41–43] [Figure 3(a)] and the two-stage opamp [22,44,45] [Figure 3(b)] are the most widespread topologies for the low-noise OTA. Other implementations have employed folded cascode opamps [46] [Figure 3(c)], telescopic cascode amplifiers [29,47] [Figure 3(d)], or fully-differential self-biased OTAs [48] [Figure 3(e)] to reduce the power overhead and noise coming from the second stage of an OTA. Moreover, the capacitive network-based topology proposed in [46], the schematic of which is depicted in Figure 3(c), improves the NEF by means of source degeneration resistors employed in the active load of the differential pair M1-M2. Using a source-degenerated load (M5-M6 and R1-R2) instead of a regular active load allows to make the bias current of the transistors in the folded branch a small fraction of the current in the differential pair, making the noise contribution of this branch negligible compare to that of M1-M2.

Moreover, the noise contribution from the source-degenerated load comes mainly from the degeneration resistors, which noise is essentially thermal, whereas MOSFETs in a regular active load contribute a large amount of 1/f noise. Table 2 summarizes the design parameters of low-noise OTA topologies. Expressions are given for the open loop gain and for the input referred noise density. The open loop gain of an OTA is obtained by calculating the product *G_m_R_o_*, where *G_m_* and *R_o_* are the overall transconductance and the output resistance of the OTA, respectively. The Miller OTA and the telescopic cascode OTA yield smaller noise densities than other topologies as fewer transistors in their implementation contribute to the total noise.

Besides, achieving small silicon area in a neural amplifier is becoming critical as the number of recording channels is continuously growing. However, a capacitive feedback network can often be area consuming. An alternate solution to such topology is proposed in [49]. This design [Figure 2(b)] employs an active feedback loop implemented with a Miller integrator using a MOS-bipolar element and a small-integrated capacitor to cancel any systematic offset and drift at the electrode-tissue interface. In addition to providing a competitive NEF and consuming low-power, such an approach enables a very small chip area since it is using only one small integrated capacitor compared to two big plus two smaller integrated capacitors needed for the capacitive-feedback network-based configuration.

Adapted versions of the neural amplifier designs introduced above are widely used to extract lower-frequency signal modalities such as EEG and LFPs [37]. In addition to that, some designs are employing active approaches like Chopper stabilization to attenuate 1/f noise [50,51], which is dominant in the low-frequency range (between 1 mHz and a few hundred Hz) for CMOS integrated devices. Besides, circuits that allow capturing multimodal information have been demonstrated. Such amplifiers can select between several types of biopotentials by accommodating different frequency ranges [18,34,44,46,52]. Different bandwidth settings can be obtained by: (1) tuning resistive values in a filter, (2) selecting different capacitors values from an array, or (3) changing the operating points of a circuit. In several designs, the high-cutoff is changed by varying the gate voltage of a pseudo-resistor [46,53], or that of a weakly inverted MOSFET [40,54]. In contrast, the low-cutoff is changed by varying the operating point of the low-noise OTA directly [37], or by changing its capacitive load through the selection of different output capacitors [55].

However, most of these designs present significant distortion and lead to variation of the high-cutoff, since the resistance of the MOS devices is highly dependent on the voltage level of the output signal. A MOS resistor exhibits asymmetric and nonlinear resistance when the voltage across it varies. It is also highly dependent on process variations. Approaches have been proposed to linearize MOS resistors by implementing dedicated linearization circuit performing appropriate biasing of the gate of the MOS devices [34,56]. Such approaches have been extended further in [57] by replacing the current mirrors and the source followers by closed-loop opamps, which allow reaching high-linearity above 74 dB within ±200 mV, and enabling process-independent frequency cut-off values. In this work, the pseudo-resistor of a lowpass filter is linearized by reporting any voltage variations in the input voltage right at its gate, with unity gain. This maintains the gate voltage of the pseudo-resistor at a constant value, cancelling any non-linearity, while providing adequate DC biasing to set the desired cut-off frequency in a preferred range. A Switch-capacitor neural amplifier with tunable characteristics was recently demonstrated in [58] as an alternate means for multimodal measurements. In addition to provide low-noise and satisfactory gain, this amplifier can accommodate LFPs and APs through tuning of its clocking frequency, implementing different low- and high-cutoff frequencies in a straightforward fashion.

On the other hand, assessing neurochemicals requires sensitive potentiostat circuits that can measure very small redox currents. Since the typical concentrations of neurotransmitters are of the order of nanomolar and lower, a potentiostat needs to measure currents on the order of nanoamperes down to picoamperes. The role of the potentiostat is to measure a current at a fixed potential. Such a system functions by maintaining the potential at the working electrode at a constant level, with respect to the reference electrode, by forcing a current through the counter electrode. In addition, a potentiostat must provide high gain, excellent linearity and wide dynamic range in order to associate a small redox current with a concentration by following a linear relationship. Integrated potentiostats have been demonstrated for the measurement and the detection of neurochemical species, and they were integrated in multi-channel system formats. Early implementations employed direct current to voltage converters made from opamps and resistor networks [35,59]. In other designs, the input current is integrated on a capacitor and the output voltage is sampled across it [60]. Recent designs are employing a delta-sigma modulator (ΔΣM) to directly convert the analog input current into a serial bit-stream. With the action of oversampling, a ΔΣM modulator reliably converts the small redox current directly into digital data with high resolution, without the need for additional building blocks performing current amplification. A switched-capacitor first-order single-bit ΔΣM is implemented in [36] for performing amperometric measurements. Such a potentiometric system provides sensitivity down to 100 fA for a power consumption of 3.4 μW per channel. It has latter been integrated in a 16-channel microsystem [25]. A continuous-time current-input second-order ΔΣM is presented in [24]. Such a potentiostat allows measurements in both FSCV and amperometry modes, and features a frequency-shift-keying transmitter operating near 433 MHz for transferring the measured data to a base station at 1.8 Mb/s.

## 5. On-Chip Data Management

**Figure 4.**
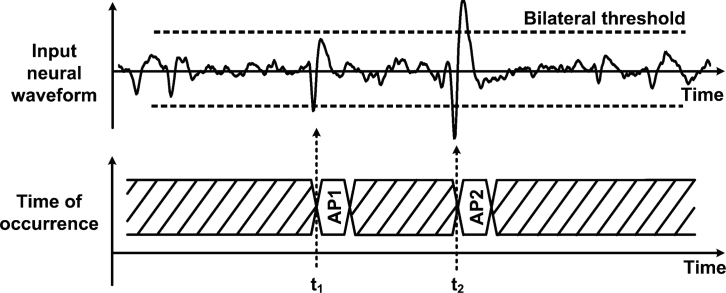
A data reduction scheme employing a bilateral threshold function. In such scheme, action potentials are detected upon threshold crossing and then tagged with their respective time of occurrence.

Data management strategies have been introduced as a means to reduce data transfer rates and power consumption that would otherwise be prohibitive in neural recording microsystems. In such schemes, on-chip circuitry performs the identification and the extraction of the incoming neural waveforms, so the system only focuses on relevant portions instead of the entire raw neural signal. Such an approach offers huge savings opportunities since neural recordings present very low duty cycles in the range of 2% to 20% [31,61], representing a maximum reduction factor of 50. Applying such a huge reduction factor results in a data rate of only 6 kbit/s per channel, assuming ideal signal detection rate. Hence, this would drastically increase the channel count from 16 channels to nearly a thousand in some proposed wireless telemetry links. When detecting a neural event, it is essential to capture multiple waveshape features in order to further utilize the extracted biopotential in subsequent processing steps, like in waveforms sorting. For instance, the time of occurrence, the maximum amplitude value and the minimum amplitude value are essentials waveform features that need to be known in order to associate a biopotential with a specific neuron. Indeed, it is worth mentioning that the best performance arises when complete waveshape representations are available [62,63]. Data management is usually performed after passing the signal into the neural amplifier, so it occupies a larger voltage range and it is less sensitive to noise. Automatic biopotential detectors that locate and extract neural waveforms in real time have been demonstrated. A simple and inexpensive scheme consists in passing the signal through a unique threshold detector for determining the biopotentials locations [64,65]. Detection occurs when the biopotentials amplitude crosses a specified voltage threshold. Implementing such scheme is straightforward and represents very low overhead, both in digital and analog formats. However, it can only measure the portion of an AP that is above the positive threshold, losing the major portion of the waveform that is below the threshold value. Moreover, accurate detection using this scheme is only possible for high signal-to-noise ratios (SNRs) [66]. Indeed, the threshold value must be carefully chosen for optimizing the detection rate according to the level of noise measured in the channel. Such noise comprises background neural noise, Johnson noise and 1/f noise. A similar, but more effective technique, consists in using a bilateral threshold to cover both positive and negative signal polarities [31,44,47,67,68]. Such bilateral threshold function along with basic feature extraction is illustrated in Figure 4.

**Figure 5.**
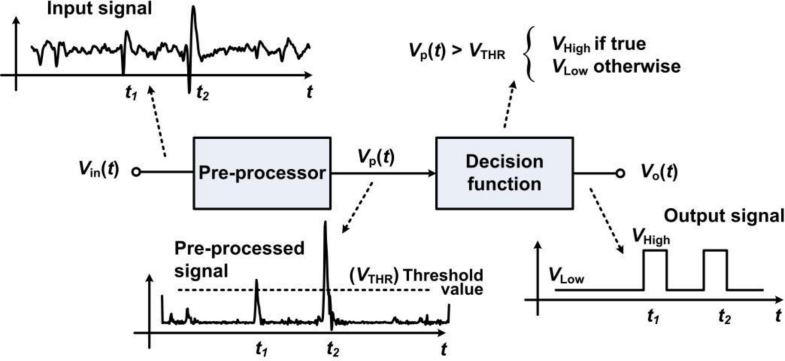
Block diagram of a signal detector employing a pre-processor.

For instance, the bilateral threshold-based detector in [31] allows capturing complete waveshapes by means of SRAM data buffering blocks. Data buffering allows capturing the incoming waveform samples ahead of detection, which is preventing the system from truncating any biopotential. Indeed, using a bilateral threshold is equivalent to employing a squared-signal pre-processor prior to a unique threshold detector. Pre-processors are additional building blocks that are employed in the detector to improve the SNR before passing the transformed waveform into the threshold function. Such pre-processor can increase the detection rate by accentuating the waveshapes and attenuating the noise for achieving high detection rate under low SNR. Figure 5 illustrates the principle of a pre-processor based detector. Several types of pre-processor-based detectors have been implemented in analog and mixed-signal formats. The detector in [54] can locate APs and measure the peak-to-trough differences, which are employed as a means for basic on-chip feature extraction. The circuit proposed in [69] employs two lowpass filters, one generating a moving average and a second filtering the input signal, to create a dynamic thresholding system. Such a design employs current-mode circuits with subthreshold-operated transistors presenting sub-microwatt energy consumption. A variance-based pre-processor detector and sorting system is proposed in [70]. An energy-based pre-processor implemented within low-power current-mode circuits in [71] allows superior detection rates, while occupying a small chip area and consuming low-power, below 1 μW. This latter design was further augmented with a dual-threshold decision function in [72].

Adaptive thresholds are often employed in the decision function of a detector to maximize the detection rate [69,73–76]. In such schemes, the value of the detection threshold is dynamically adjusted based on the SNR of the input signal and its background noise. An analog implementation of such scheme is proposed in [74]. In this work, a low-power subthreshold-based current-mode local averaging filter is employed to adaptively adjust the value of the detection threshold represented by a voltage in the decision function.

Digital signal processors have also been implemented to perform detection, feature extraction, data compression, as well as spike sorting. A wavelet-based digital processor based on a lifting scheme allows the detection and the compression of 32 channels of input neural data [77]. This processor implemented in a 0.18-μm CMOS process consumes 76 μW and presents a chip area of 0.22 mm^2^. A polyphase-based discrete-time wavelet processor is proposed in [78]. This latter work is also covering the implementation of continuous-time wavelets in hardware. A real-time neural spike sorter based on a low-complexity and adaptive digital neural signal processor is employed in [79] to minimize the bandwidth and the overall power dissipation in a neural recording microsystem. Such a processor extracts basic AP parameters such as the Euclidian distance, frequency of spike occurrences and the total number of spikes, and stores such information in a cache entry. A template-matching algorithm allows assigning the detected APs to specific clusters for speeding up the classification of waveforms off the chip. Similarly to a CMOS imager, a switched-capacitor delta read-out data compression circuit is employed in [80] to compress neural waveforms related to epileptic seizures. By trading recording accuracy for the output data rate, this system allows compression factors as high as 800. A digital energy-based processor is employed in [55] to perform on the fly-feature extraction in a 128-channel neural recording microsystem. Such a processor allows identifying spikes and processing them with a digital frequency-shaping filter. Such an operator is capturing a subset of samples containing the necessary information for clustering the data off the chip, and reducing the payload that has to be transmitted via a RF link. The processor can handle a single channel among 128 and consumes 0.1 mW.

## 6. Smart Power Scheduling

Power scheduling mechanisms are employed to decrease the power consumption in dense implantable microsystems featuring power hungry modules such as low-noise sensory circuits, data converters or data transmitters. Such approaches consist in powering up the building blocks only when necessary. Current-supply modulation [34,43] and duty cycling [34,55,57] are power scheduling techniques consisting in switching specific building blocks between an active and an idle state with low duty cycle. The shorter the duty cycling period is, the lower the power dissipation in the circuits is. The difference between both techniques is that the former uses low-level bias current in the idle state, whereas the latter has zero current in such state for further increasing power savings. A duty cycling period that is too short can potentially degrade in the reading accuracy of a neural recording channel, because fast intermittent monitoring requires circuits featuring larger bandwidth, thus letting more noise entering. The battery-powered sensor interface presented in [57] addresses this tradeoff by providing different levels of accuracy and lifetime through the utilization of a programmable low-pass filter. Such a filter allows selecting between different input-referred noise levels and duty cycle lengths, which determines the overall accuracy and power consumption. The power-scheduling mechanism employed in [34] consists in putting most of the neural amplifiers that are not being sampled in sleep mode with a fraction of their active current consumption (0.5 μA). Indeed, not turning the neural amplifiers completely off lets time for the neural amplifiers to reach their active state faster ahead of each sampling. This scheme allows one to decrease power consumption by 18% in the multi-channel front-end block. Besides, the multiplexing of several electrodes towards one neural amplifier has been demonstrated as a means to save power and silicon area. Time division multiplexing of four electrodes towards a single neural amplifier is achieved in [79]. Such time multiplexed topology shares a single LNA between four independent input electrodes to decrease the power consumption per channel by one fourth. The LNA is based on a capacitive-feedback low-noise OTA featuring multiple feedback networks. Each feedback network belongs to a specific channel. During time multiplexing and throughout a sampling period, each channel operates either in track or hold modes. During tracking, a set of MOS switches connects a specific capacitive network in the feedback loop of the LNA to enable recording from an associated input channel with a closed-loop gain of 40 dB. The implementation of such a scheme results in a similar system-level architecture as shown in Figure 1(a), but with the analog multiplexer relocated before the sensory channels building blocks. A frequency-division multiplexing scheme is implemented in [81] where the amplitudes of the neural activity seen at each individual electrode is modulated and directed towards a single wideband neural amplifier. It is shown in [81] that the maximum number of electrodes that can be multiplexed towards one single amplifier is limited by the summation of the thermal noise from each site at the input node of the wideband neural amplifier, which is in the range of 5 to 10 for typical cases.

Activity-based schemes exploit the transient characteristics of neural signals to maximize efficiency. Adaptive sampling [82,83] is an activity-based technique that allows a significant decrease in the data rate by an order of magnitude. Such an asynchronous data-sampling scheme dynamically varies the sampling rate of the data converter based on the input signal activity. Reduction factors of 7 are reported with this scheme. The algorithm requires the implementation of the 2nd derivative of the signal within a dedicated circuit to measure the rate of change of the input signal. Other activity-based schemes exploit the intermittent nature of neural recordings along with their low duty cycles by powering up the building blocks only when neural events occur. Such a strategy requires the utilization of accurate signal detectors along with a power scheduling strategy. In such schemes, most building blocks remain in an idle state between occurrences of neural events, draining practically no power. In [84], a low-overhead analog detector is employed to locate neural events and triggering up the recording electronics. Indeed, the approach proposed in [61] is using low-overhead analog delay elements that are implemented within ultralow-power linear-delay filters in order to waking up the recording circuits “ahead” of biopotential occurrences, whose functionality is critical to avoid truncated waveforms. Adaptive mechanisms have been proposed for achieving high-power efficiency. In such approaches, the DC operating points of a circuit are adjusted dynamically by employing feedback. A neural amplifier using adaptive biasing is demonstrated in [85]. Such closed-loop scheme is directly adjusting the SNR of an LNA by changing its bias current in order to set the input referred-noise of the amplifier right above the noise floor of the input electrode, which scheme is avoiding any waste of energy.

## 7. Low-Power Telemetry and Wireless Power Supply

In contrast with head mounted research devices that operate externally to the body, most implantable microsystems cannot use batteries as primary sources of energy. Instead, power must be delivered wirelessly across the skin through an inductive link formed by a pair of coils. Inductive coupling is among the safest method to power up implants because it avoids the biological risks and encumbrance associated with transcutaneous wires. In such a link, a power amplifier drives an external coil (the primary) to deliver energy to an implanted coil (the secondary) through inductive coupling. A voltage rectifier and a voltage regulator follow the secondary for delivering an adequate supply voltage and current to the implanted circuits. This concept has been demonstrated in several applications [86–89]. Maximizing the wireless power transfer efficiency is critical to reduce the size of the energy source, heating of the tissues and interference with other devices. However, the effect of the surrounding environment and the presence of parasitic components are known to largely influence the resulting efficiency [90]. On the other hand, resonance-based wireless power delivery has recently received a lot of attention. This efficient approach allows one to transfer power over longer distances using a four-coil coupling system in which the adverse effects resulting from a low coupling coefficient between the primary coil and the secondary coil can be compensated by using high-quality factor coils [87].

**Figure 6.**
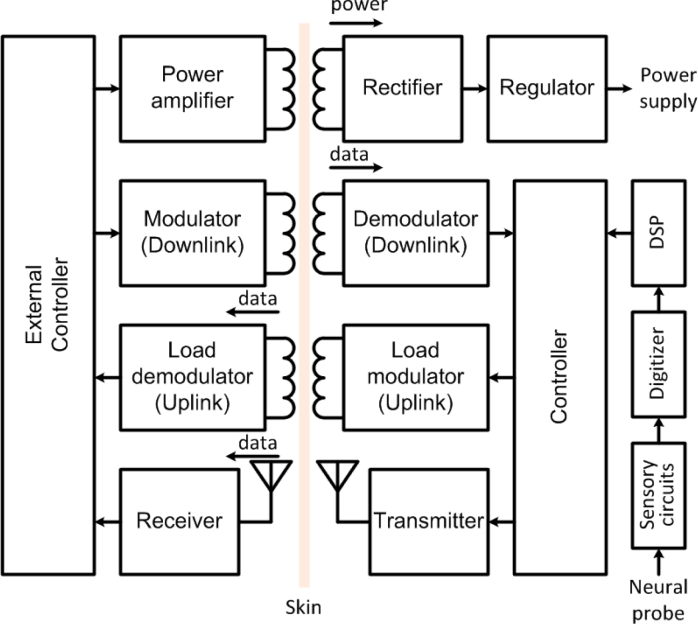
Block diagram of an implantable neural recording microsystem and its multi-carrier wireless interface.

Wireless telemetry has traditionally been combined with a wireless power supply on a same near-field inductive link using amplitude-shift-keying (ASK) or on-off-keying (OOK) modulation schemes for the uplink, while using back telemetry through Load-Shift-Keying (LSK) modulation for the downlink [85,88,91]. In back telemetry, the variations induced in the load at the secondary coil (implanted) are sensed at the primary (external) coil by a controller. Data rates up to 5.8 Mbps have been reported with such a technique [85]. Later, power and data have been separated in multiple independent links using multi-carrier approaches [23,32,92,93]. Such a multi-carrier interface is illustrated in Figure 6.

In such a scheme, the power link is typically operated at lower carrier frequency (at a few megahertz) to limit absorption in biological tissues, while maximizing power transfer efficiency, whereas the uplink and downlink are pushed at higher frequencies to increase the data rate, while minimizing crosstalk and interference. In accordance with such a design philosophy, there is a high interest in low-power transmitters that stream data in the far field for enabling short-range monitoring at high rate. In such schemes, the uplink is operated at much higher frequencies than the power carrier and the downlink center frequency. Several neural recording devices employing RF transmitters have been developed with components off-the-shelf (COTS) [42,67,94]. For instance, HermesD is a COTS-based battery-powered neural recording system that allows the transmission of 32 channels of neural data using frequency-shift keying (FSK) modulation, with an adjustable carrier frequency between 3.7 to 4.1 GHz [94]. A COTS transmitter allows a data rate of 24 Mb/s within a range extending over 20 m. In [67], a COTS transmitter is transferring 96 channels of neural data from a single chip neural recording microsystem over a range of 2 m, using amplitude-shift-keying (ASK) modulation. But, despite the high data rate achieved, the significant power consumption of these COTS transmitters (>100 mW) prevents their utilization in fully implantable or battery-less systems.

Custom integrated transmitters have been designed for operating within a power budget below 10 mW. Early integrated wireless transmitters have employed analog modulation schemes. In [95], frequency-modulation (FM) is employed for transmitting the neural activity from a 4-channel neural microsystem in the band of 94–98 MHz over a distance of 0.5 m. Wireless EEG recordings were performed in untethered rodents with an inductively powered neural microsystem employing an FM transmitter featuring a 3.2 GHz carrier [96]. The utilization of neural microsystems in sensitive locations prompted the implementation of low-power biotelemetry systems that comply with international regulations. Such transmitters preferably operate in the low-overhead unlicensed ISM bands, with center frequencies of 433.92 MHz, 915 MHz and 2.45 MHz, or in the MICS band between 402 MHz and 405 MHz, specifically defined for bi-directional communication with medical implants. This latter band allows a maximum transmitted power of 25 μW. An integrated FM transmitter operating near 433 MHz and consuming less than 0.5 mW is reported in [97] for transferring a single recording channel. More recently, several low-power integrated RF transmitters employing digital modulation schemes have been reported to transmit bioelectrical and neurochemical activity from multiple channels [24,28,32,34,47]. Such RF transmitters typically employ frequency-shift-keying (FSK) and can achieve data rates reaching 10 Mb/s. They normally require the implementation of voltage-controlled oscillators, a power amplifier and an LC tank that drives a small antenna. The 100-channel system reported in [28] employs a 902–928 MHz FSK transmitter achieving a data rate of 345.6 kb/s within a range of a few centimeters, without using a transmitting antenna. This microsystem is inductively powered from a 2.765 MHz carrier and a microfabricated printed coil for the secondary. In [34], a head-mounted recording system dedicated to research applications is inductively powered at 13.56 MHz and is transmitting a 915-MHz FSK-encoded asynchronous PWM-TDM signal. This system allows transferring 32 channels of multiplexed neural data at a maximum rate of 710 kS/s within 1.2 m of range, and it employs a miniature wideband monopole transmitting antenna. This latter system enjoys a digitizer-less architecture avoiding the need for an on-chip data-converter, thus saving considerable power. Besides, the reader must note that these works are reporting in-air performances.

In contrast with carrier-based transmitters, carrier-less transmitters that rely on edge-combining frequency multiplication have been demonstrated. A sub-milliwatt MICS-band and 433-MHz ISM-band compliant transmitter using such a scheme is presented in [98]. This transmitter provides 100 kb/s for a consumption of 0.4 mW. Its operating principle is based on a delay locked loop and edge combining frequency multiplication. A 405-MHz carrier frequency is generated by combining nine equally spaced edges of a 44.6-MHz clock provided by a crystal reference oscillator. The edge combiner behaves like a non-linear power amplifier that drives an off-chip LC tank matching an antenna. Besides, a novel carrier-less near-field telemetry modulation scheme is proposed in [99]. Pulse Harmonic Modulation (PHM) is a novel wideband modulation technique for achieving high data rate transmissions over inductive links. Data rates above 5 Mb/s are reported with this technique.

On the other hand, ultrawide-band (UWB) communications in the regulated 3.1-to-10.6 GHz band are actively researched as a low-power wireless short-range technology, enabling data rates of several tens of Mb/s [100]. Unlike conventional narrow-band communications, impulse-radio (IR) UWB uses short-duration pulses in the time domain to convey information, which promotes a low-duty cycle, low interference, and better immunity to multipath fading. Two main techniques are employed for pulse synthesis. A first architecture consists in generating a pulse at baseband and up-converting it in the UWB band using a mixer and a local oscillator [101]. A second architecture consists in generating pulses that directly fall into the UWB band by using pulse combination, without any up-conversion, similarly to the case of carrier-less narrow-band transmitters [102]. A passive off-chip LC filter can be employed to shape the output spectrum of the transmitter according to the regulation for indoor UWB emission. Such pulse shaping approach has allowed data rates up to 15 Mb/s [103] and 90 Mb/s [55], for power consumption of 4.85 mW in a 0.5-μm CMOS process, and 1.6 mW in a 0.35-μm CMOS process, respectively. Modulation schemes such as OOK or Pulse-position modulation (PPM) are typically employed for low-power UWB communications. Carrier-less IR-UWB transmitters can have simple architectures, allowing low-power and small chip area in CMOS technology, whereas complex receivers can be easily built using COTS, since the power budget and the footprint of the remote base station is not crucial.

Besides, optical telemetry is employed for the uplink in [14,33] as an effective means to achieve high data rate and low interference. Such multi-modal wireless interface is based on an embedded high-efficiency vertical-cavity surface-emitting laser diode allowing the transmission of the neural data that are measured on 16 channels. This head-mounted recording system is inductively powered and achieves a peak optical output of 2 mW. This microsystem is consuming a total power of 12 mW and is currently being used to collect *in-vivo* data from the primary motor cortex of behaving rhesus monkeys. Moreover, microwave-based energy recovery has been proposed for a wireless switch circuit dedicated to an implantable endoscope capsule in [104]. In such design, a multi-stage rectifier is used to convert the received 915 MHz RF power into a DC voltage supply stored on a 300 pF on-chip capacitor. A simple voltage regulator following the rectifier is used to stabilize this DC voltage supply and retrieve a few micro-amperes, which is limiting such an approach for utilization in very low supply current applications.

## 8. Conclusions and Outlook

While wireless microsystems have been used to successfully retrieve the neural data in untethered animals, there are still several technical issues that need to be addressed before development of clinically viable prosthetic devices or mature research applications will be possible. The challenges fall into two categories. First, the next generation of neural devices will need to provide a better reliability and long lifetime for enabling safe chronic utilization. Long-term stability requires microsystems achieving perfect biocompatibility. In such case, the interaction with the biological tissue layers must be minimized in order to avoid any foreign body responses, like adhesion with the dural tissue [31]. Designing better coating materials and new hermetical sealing techniques is of great interest. Secondly, neural interfacing devices are brought to provide increasing resolutions and multi-modal sensing. In fact, reaching better resolution and higher quality of data across multiple realms, and with different signal modalities, is an important milestone. Thus, the next generations of neural microsystems may certainly need to cope with overwhelming densities, requiring novel approach and techniques for meeting the requirements. Reaching high power efficiency in implantable ICs will remain an important issue.

Besides, neural recording devices will need to provide accurate selectivity to isolate a given signal modality or to discriminate between specific responses from the CNS. Such flexibility is a requisite for addressing several modalities in a simultaneous fashion, and for extracting a relevant information content on-chip to reduce a complex dimensionality into a small data set that can be handled by low-power telemetry. The implementation of such functions will require significant advances in the design of dedicated sensory circuits and in the implementation of efficient signal processing algorithms.

The design of low-power miniaturized biotelemetry transmitters is of constant relevance. Future systems will need to address sensitivity issues related to interference, component parasitics and surrounding biological tissues in near-field and far-field wireless links. Moreover, as the complexity of microsystems is scaled up while their size is scaling down, an important issue will arise from the fact that implantable devices may come to a point where they will need much more energy within a much smaller footprint. A solution to such an issue consists in diversifying power sources. For instance, photovoltaic energy converters are used in combination with inductive power delivery in [14,105].

In addition, designing high-resolution microsystems providing a bidirectional access to several individual neurons of the cortex through electrical stimulation and neural recording capabilities in the same device is challenging, but very much needed. Such systems will render it possible to interact with groups of neurons in closed loop, *i.e.*, to use electrical stimulation in conjunction with neural recording to activate specific neural networks and retrieve instantaneous feedback information on their status. Having access to such bidirectional neural interfaces will have considerable impact on experimental brain research and on the development of new assistive technologies to treat chronic diseases and neural disorders like Parkinson, epilepsy, dystonia and dysphasia. Moreover, combining neurochemical sensing, drug delivery and microfluidics together within such bidirectional interfacing device is among future challenges. The development of alternate neural recording and microstimulation approaches should also attract more attention. For instance, an imaging-based neural recording technique in presented in [106].

In conclusion, neural interfacing technology has recently seen tremendous progress, but its development into viable clinical systems still faces important technological challenges. There are presently several open multidisciplinary directions that are being investigated for subsequent development.

## Figures and Tables

**Table 1.** Summary of Neural Recording Parameters for Different Signal Modalities.

**Neural signal modality**	**Measurement technique**	**Amplitude**	**Bandwidth**	**Electrodes**	**Potential for chronic utilization**
**Extracellular action potentials**	Voltage amplification	50 to 500 μVpp	100 Hz to 10 kHz	Metal/silicon microelectrode	High
**Intracellular action potentials**	Voltage amplification	10 to 70 mVpp	100 Hz to 10 kHz	Glass micropipette	Very low
**Local field potentials**	Voltage amplification	0.5 to 5 mVpp	1 mHz to 200 Hz	Metal/silicon microelectrode	High
**Electroencephalogram**	Voltage amplification	1 to 10 mVpp	1 mHz to 200 Hz	Surface electrode	Very high
**Ionic current**	Patch clamping	1 to 10 nA	1 mHz to 10 kHz	Glass micropipette	Low
**Redox current**	Amperometry FSCV	100 fA to 10 μA	1 mHz to 100 Hz 1 mHz to 10 kHz	Iridium oxide/carbon fiber microelectrode	High

**Table 2.** Summary of Low-Noise OTA Parameters ^1^.

**Opamp topology**	**Open loop gain (*G_m_R_o_*)**	**Input-referred noise density**
**Current-mirror OTA [Figure 3(a)]**	$g_{m1}(g_{m10}r_{o10}r_{o8}∥g_{m9}r_{o9}r_{o6})$	$\frac{16kT}{3g_{m1}^{2}}(g_{m1}+2g_{m3}+g_{m7})$
**Miller OTA [Figure 3(b)]**	$g_{m1}(r_{o2}∥r_{o4})\cdot g_{m7}r_{o7}$	$\frac{16kT}{3g_{m1}^{2}}(g_{m1}+g_{m3})$
**Folded OTA with source degeneration [Figure 3(c)] ^2^**	$\begin{array}{l} g_{m1}\alpha (g_{m8}r_{o8}\cdot g_{m6}r_{o6}R_{2})\\ \;\;\;\;\;\;\;\;\;∥(g_{m10}r_{o10}r_{o12})] \end{array}$	$\frac{16kT}{3g_{m1}^{2}}(g_{m1}+\frac{2}{R_{1}}+g_{m11})$
**Telescopic OTA [**Figure 3(d)]	$g_{m1}(g_{m3}r_{o1}r_{o3}∥g_{m5}r_{o5}r_{o7})$	$\frac{16kT}{3g_{m1}^{2}}(g_{m1}+g_{m7})$

1 Note that *g_m_* and *r_o_* represent the transconductance and the output resistance of a MOS device, respectively, while *k* is the Boltzman constant and *T* is the temperature.

2 Parameter *α* in the open loop gain of the Folded OTA depends on the impedance looking into the sources of M7-M8 and can be calculated as detailed in [46].
